# Axial Attention Convolutional Neural Network for Brain Tumor Segmentation with Multi-Modality MRI Scans

**DOI:** 10.3390/brainsci13010012

**Published:** 2022-12-21

**Authors:** Weiwei Tian, Dengwang Li, Mengyu Lv, Pu Huang

**Affiliations:** 1 Shandong Key Laboratory of Medical Physics and Image Processing, Shandong Institute of Industrial Technology for Health Sciences and Precision Medicine, School of Physics and Electronics, Shandong Normal University, Jinan 250358, China; 2 School of Environment and Energy, South China University of Technology, Guangzhou 510006, China

**Keywords:** MRI, brain tumor segmentation, attention mechanism, deep learning, deep supervision

## Abstract

Accurately identifying tumors from MRI scans is of the utmost importance for clinical diagnostics and when making plans regarding brain tumor treatment. However, manual segmentation is a challenging and time-consuming process in practice and exhibits a high degree of variability between doctors. Therefore, an axial attention brain tumor segmentation network was established in this paper, automatically segmenting tumor subregions from multi-modality MRIs. The axial attention mechanism was employed to capture richer semantic information, which makes it easier for models to provide local–global contextual information by incorporating local and global feature representations while simplifying the computational complexity. The deep supervision mechanism is employed to avoid vanishing gradients and guide the AABTS-Net to generate better feature representations. The hybrid loss is employed in the model to handle the class imbalance of the dataset. Furthermore, we conduct comprehensive experiments on the BraTS 2019 and 2020 datasets. The proposed AABTS-Net shows greater robustness and accuracy, which signifies that the model can be employed in clinical practice and provides a new avenue for medical image segmentation systems.

## 1. Introduction

**Figure 1:**
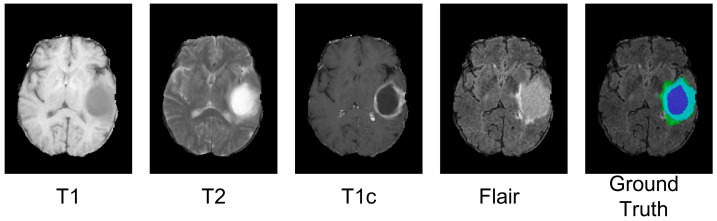
A visualized case is chosen from the BraTS2021 training dataset. (Different colors present different tumor types. Whole Tumors (WTs) are shown in blue, purple, and green, Tumor Cores (TCs) are shown in blue and purple and Enhanced Tumors (ETs) are shown in blue.)

Brain tumors, defined as abnormal cells that grow and multiply uncontrollably inside the brain, are not only dangerous to health but also lead to death as the tumor spreads malignantly. Brain tumors can be grouped into secondary and primary forms [1]. The former refers to tumors that grow elsewhere in the body and metastasize to the brain; the latter refers to tumors that originate from the brain tissues or the immediate surroundings of the brain and have a higher incidence. Glioma, the most prevalent primary cancer, accounting for 78 percent of malignant brain tumors, is usually treated with surgical resection, radiotherapy, and chemotherapy. The World Health Organization (WHO) classifies gliomas into two categories: low-grade gliomas (LGG) and high-grade gliomas (HGG), including grades I to IV [2]. Grade I refers to benign tumors that can be resected from the skull and are curable. Grades II and III gradually become malignant, while grade IV refers to highly malignant tumors with the worst prognosis. Early detection and diagnosis of gliomas can help experts to develop accurate treatment plans to prolong survival. Magnetic resonance imaging (MRI) [3] is the examination method of choice for the diagnosis and treatment of gliomas, mainly due to its presenting detailed brain structural information by enabling tomography in any direction and generating non-invasive multi-modality images [4]. Four MRI modalities (Figure 1) are most commonly used to label brain tumors. Brain tumor segmentation aims to mark the tumor regions using an MRI of the brain, playing an essential role in clinical diagnostics. Accurately identifying tumors from MRI images relies on professional skills [5] and the experience level of radiologists [6]. Furthermore, the manual diagnosis of massive MRI images by radiologists is a time-consuming task. Therefore, an efficient and accurate automatic method is crucial for assisting doctors in segmenting brain tumors from a mass of MRI data.

Taking the above into consideration, we propose an axial attention brain tumor segmentation network (AABTS-Net) with a hybrid loss function, automatically segmenting tumor subregions based on multi-modality MRI. An axial attention mechanism (AAM) has been employed, enhancing the ability to model global dependencies. It considers the relationships of pixels to each other on different dimensions, using long-range cues to guide the segmentation while having fewer parameters and taking less GPU memory. The AAM consists of a depth self-attention module, a height self-attention module, and a width self-attention module. The depth self-attention module enhances the relationships of pixels to each other on the depth dimension. The height self-attention module and width self-attention module capture the relationships of pixels to each other on the height and width dimensions. A deep supervision (DS) mechanism is employed in the AABTS-Net, addressing the gradient vanishing, and guiding the generation of better feature representations at each layer of the network. The training loss of the network is the weighted sum of the loss of the intermediate and last outputs. The hybrid loss function is applied for the purposes of increasing the sensitivity of segmenting tumors, including a batch-based Dice loss for improving training stability on the imbalanced samples and a binary cross-entropy loss for handling issues arising from varying tumor sizes. The proposed hybrid loss function can direct our model toward paying more attention to the tumor region and achieving ideal segmentation results, overcoming the problem of tumors of different sizes and even in the case of small target tumors. Regarding segmentation performance, the proposed AABTS-Net can make reliable results on the BraTS 2021 validation dataset for segmenting three subregions (ET, TC, and WT), achieving 0.830, 0.861, and 0.922 in DSC, respectively. Furthermore, we conducted comparative experiments involving other state-of-the-art methods on the BraTS 2019 and 2021 validation datasets, showing that our method had greater robustness and accuracy.

Specifically, the main contributions of this study include:

(1) Applying AAM to improve segmentation accuracy makes full use of the relationships of pixels on different dimensions and enhances the ability to model global dependencies. Notably, an axial attention mechanism, presenting a novel method to solve computational complexity, is employed to capture plenty of the semantic information that is essential for segmenting the boundary of the tumor and retaining more detailed tumor information.

(2) Handling the vanishing gradient and improving the feature expression ability of the model by applying the deep supervision mechanism on the decoder to guide the model to generate better feature representations.

(3) Suppressing the effect of the dataset class imbalance by introducing a hybrid loss function to emphasize the lesion area. The hybrid loss function can guide our model to pay closer attention to the tumor region to achieve ideal segmentation results, overcoming the problem of tumors of different sizes and even small target tumors.

## 2. Related Work

### 2.1. DL-Based Methods for Brain Tumor Segmentation

The convolutional neural network (CNN), an effective deep learning (DL)-based architecture, has attracted more and more attention from researchers for image-processing tasks, such as image classification and semantic image segmentation. Compared with ML-based methods, the most significant advantage of a CNN is that it automatically learns representative and complex feature representations from the raw images, forming a feature-learning model with high robustness and adaptability. A dual-path architecture, combining local and global information, was designed by Kamnitsas et al. [7] to process multi-scale images simultaneously and extend the fully connected CRF to three dimensions (3D) for handling arbitrarily large neighborhoods. The disadvantage of this method is that the post-processing process is complicated, and the accuracy will decrease after removing the post-processing operation. Havaei et al. [8] proposed a two-channel CNN for segmentation, which includes convolutional and fully connected layers. However, the method has more parameters, and the complex structure of the model makes the network hard to train. Pereira et al. [9] adopted a deeper CNN structure and multiple 3 × 3 convolution kernels to replace the 7 × 7 and 5 × 5 convolution kernels. The 3 × 3 convolution kernel was applied to improve the operation speed of CNN and enhance the ability to extract features, making the segmentation accuracy reach about 87%. Although the method simplifies the network structure, it misses a massive quantity of local–global information, leading to low segmentation accuracy. Zhao et al. [10] used image slices with FCNN parameters to train three models and adopted a voting-based strategy to fuse the three models for final segmentation. Chen et al. [11] introduced an FCNN-based multi-scale receptive field and a densely connected blocks-based hierarchical architecture to consider the different types of brain tumors, using a block-level training strategy to alleviate the class imbalance. The two-stage method alleviates the class imbalance, while the segmentation accuracy shows no significant improvement. CNN, a widely adopted architecture for segmentation, only obtained limited accuracy improvement due to the smaller training dataset available. To address this problem, Ronneberger et al. [12] proposed the U-Net via modifying and extending the FCN, enabling it to use fewer training images and produce more accurate segmentation. Wang et al. established a 3D U-Net-based model using the strategy of brain normalization and patching [13], achieving 0.737, 0.807, and 0.894 in terms of DSC, respectively. To reduce computational complexity and enhance feature representation, Cheng et al. [14] further proposed MECU-Net by applying multi-scale feature fusion modules, fewer down-sampling channels, and cascaded architecture to 3D U-Net. Chen et al. proposed a separable 3D U-Net architecture using separable 3D convolutions [15]. Jiang et al. [16] proposed an end-to-end two-stage cascaded U-Net architecture to segment brain tumor substructures from coarse to fine and won the first prize in the BraTS2019. These methods show the effectiveness of the U-shaped structure; therefore, we chose the U-shaped structure as our network framework.

### 2.2. The Attention-Based Module for Brain Tumor Segmentation

Various attention-based modules have been proposed by researchers, facilitating segmentation accuracy by enhancing the ability to identify relevant feature representations. Zhou et al. [17] applied an attention mechanism to the encoder–decoder structure to fuse features from cross-modality to emphasize the most closely related features. Xu et al. [18] proposed a CH-UNet for brain tumor segmentation by applying a corner attention module (CAM) and high-dimensional perceptual loss (HDPL) to U-Net. The CAM can capture rich contextual dependencies and extract complementary inter- and intra-slice information. The HDPL preserves local consistency and explores perceptual similarities to fine the predicted boundary. These two methods show that the attention mechanism plays an important role in focusing on task-related features. Zhang et al. combined the residual module and attention gate with the original U-Net architecture [19] to extract rich semantic information. However, this method ignores the local–global information and leads to poor performance. Mazumdar et al. proposed an efficient spatial attention (ESA) block containing a depth-wise separable convolutional layer and a lightweight spatial attention module, using it to build an efficient spatial attention network (ESA-Net) [20], an improved variant of the popular U-Net. Kong et al. proposed a 3D, fully convolutional network (FCN) with a dual attention mechanism [21], segmenting different gliomas simultaneously. The multi-inception residual attention U-Net (MIRAU-Net), developed by AboElenein et al. [22], is based on an encoder–decoder architecture. Inception Residual is applied to connect the encoder–decoder, to reduce the distance between their feature maps for further enhancing segmentation performance. However, due to the limitations of convolution operations, CNN-based attention has a weak ability to learn global dependencies, resulting in poor performance in the context of brain tumor segmentation. To solve the problem, Wang et al. proposed a novel TransBTS, based on the encoder–decoder structure [23], applying a transformer to 3D CNN, improving the performance by combining the CNN and transformer while exploiting and modeling local–global information. In addition, Jia et al. proposed a combined CNN-transformer model called BiTr-UNet, with specific modifications [24] for multi-modality MRI segmentation. Although transformer-based models can capture global correlation, transformer-based models are not kind to GPU memory. Moreover, the computational complexity of using transformers is relatively high when handling 3D medical images. Hence, we propose an efficient AABTS-Net, which combines axial attention and CNN to automatically segment tumor subregions by taking into account the required computational complexity.

## 3. Methodology

### 3.1. The Structure of the AABTS-Net

**Figure 2:**
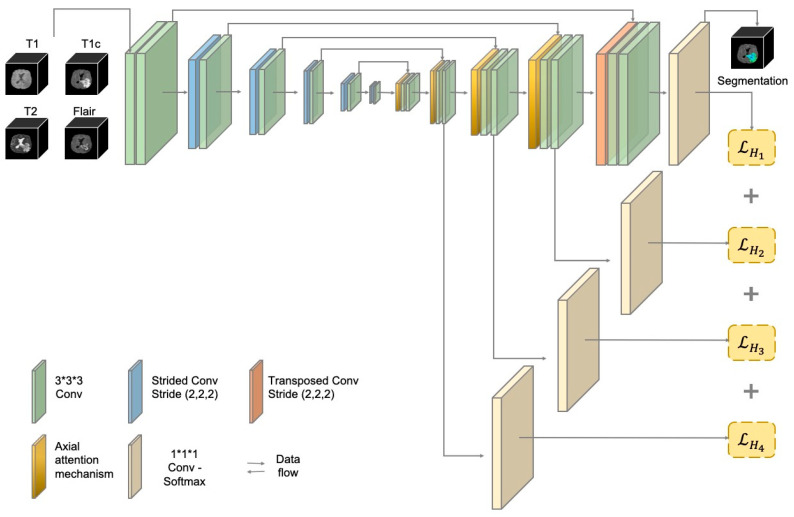
The structure of the proposed AABTS-Net.

The suggested U-shaped encoder–decoder structure with fewer parameters shows good segmentation performance in many image segmentation tasks. Similar to the 3D U-Net (39), three-dimensional operations were employed in the AABTS-Net (Figure 2), focusing on detailed and local feature representations that are suitable for brain tumor (small object) segmentation. In detail, AABTS-Net consists of an encoding path, a decoding path, and skip connections. The encoding path, including five basic convolutional blocks and five down-samplings, is used to extract feature representations at different resolution levels and reduce the resolution of feature representations, respectively. The basic convolutional block contains two consecutive convolution layers, applying Leaky ReLU (LReLU) with a slope of 0.01 as the activation function after each convolution operation. Stridden convolution operations have been considered a popular method applied in the encoding path for down-sampling. The initial size of the feature representation is 128 × 128 × 128, and the resolution of the feature representation doubles for each down-sampling operation, with a minimum of 4 × 4 × 4. The initial number of filters is 32, and the number of filters doubles with each down-sampling operation, up to a maximum of 320. A skip connection is employed to fuse feature representations from the same resolution levels of the encoding and decoding paths. Similar to the encoding path, the decoding path consists of five basic convolutional blocks and five up-samplings that employ transposed convolutions to restore the resolution of the feature representation. The sigmoid activation has replaced the softmax nonlinearity of the last layer. The input requires four-channel 3D images (4 × 128 × 128 × 128) merged by four modalities, while the output comprises three-channel predicted segmentation maps (3 × 128 × 128 × 128).

### 3.2. The Axial Attention Mechanism

As an effective modeling method for capturing global context information, global feature interaction is one of the greatest advantages of self-attention [25]. The self-attention module captures global context information, resulting in a large memory capacity and high computational complexity. When the self-attention mechanism is applied to three-dimensional data, it shows higher computational complexity. Most previous attention modules reduce the ability to model position-dependent interactions without considering the positional information that is crucial for capturing spatial structure or shape. Axial attention [26], applying self-attention to each axis of the input independently, has been proposed to reduce the computational complexity. Therefore, we employed the axial attention mechanism for three-dimensional medical image segmentation, capturing the long-term dependencies of the brain MRI and computing a representation of the local–global information. In detail, axial attention is applied independently on each axis (depth, height, and width). The AAM receives two inputs; one is the feature representation (with all contextual and spatial information) of the same resolution level, passed from the skip connection, and the other is the feature representation from the previous resolution level.

**Figure 3:**
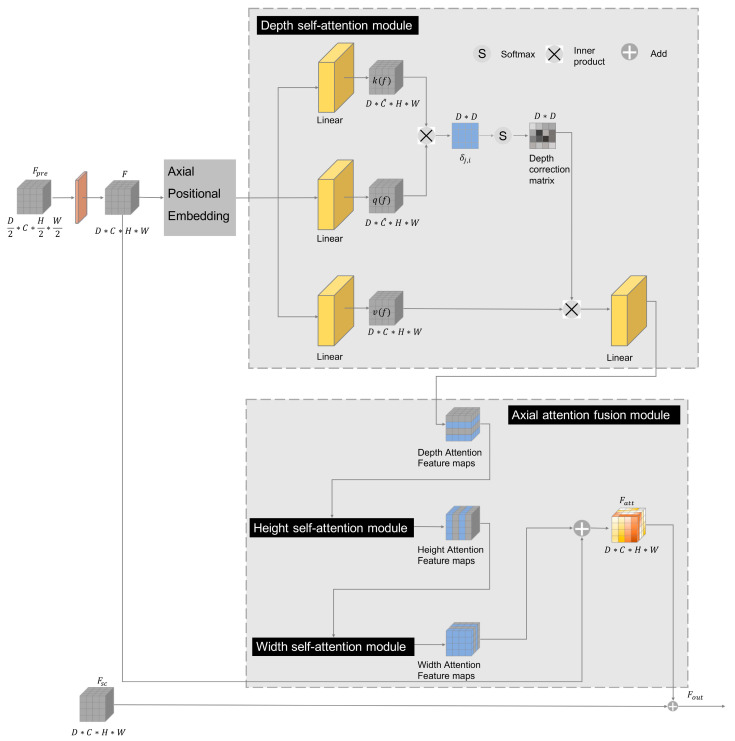
The structure of the proposed axial attention mechanism.

Figure 3 shows the AAM. Specifically, the features of the skip connection are represented as $F_{sc}\in {\mathbb{R}}^{D*C*H*W}$, where *D*, *C*, *H*, and *W* denote the depth, channel number, height, and width of the feature map, respectively. The feature of the previous layer is represented as $F_{pre}\in {\mathbb{R}}^{\frac{D}{2}*C*\frac{H}{2}*\frac{W}{2}}$. We first perform a transposed convolution on the $F_{pre}$ to obtain the feature $F\in {\mathbb{R}}^{D*C*H*W}$. With respect to self-attention, it performs the attention process with each input pixel without considering the input position information, which will lose the original position information. Therefore, we perform axial position embedding on the feature map to ensure position information. The depth self-attention module (Figure 3) is first applied to compute the attention maps representing the relationships of pixels to each other on the different slices, which calculates a similarity matrix along the depth dimension. In detail, we obtain three feature spaces $q\left(f\right)\in {\mathbb{R}}^{D*\hat{C}*H*W}$,$k\left(f\right)\in {\mathbb{R}}^{D*\hat{C}*H*W}$,and $v\left(f\right)\in {\mathbb{R}}^{D*C*H*W}$ by conducting linear operations to transform the input feature map F, which can be represented by Equations (1)–(3).

(1) $$q\left(f\right)=Linear\left(F\right)=FW_{q}$$

(2) $$k\left(f\right)=Linear\left(F\right)=FW_{k}$$

(3) $$v\left(f\right)=Linear\left(F\right)=FW_{v}$$

Here, the $W_{q}$, $W_{k}$, and $W_{v}$ are matrixes to improve the fitting ability, which can be obtained by training.

Then, q(f) and k(f) are transposed from $D*\hat{C}*H*W$ to $\left(H*W\right)*D*\hat{C}$. The inner product is employed to compute the correlation matrix, which is shown in Equation (4). The depth correlation matrix $\delta_{j,i}$ is normalized by the softmax function shown in Equation (5).

(4) $$\delta_{j,i}=q{\left(f_{i}\right)}^{T}k\left(f_{j}\right)$$

(5) $$r_{j,i}=Softmax\left(\delta_{j,i}\right)=\frac{e^{\delta_{j,i}}}{\sum_{i=1}^{N}e^{\delta_{j,i}}}$$

The deep value of $\delta_{j,i},$ with a dimension of ${\mathbb{R}}^{D*D}$, denotes the depth correlation matrix.

Finally, the depth correlation matrix $r_{j,i}$ and the v(f) were performed by the inner product to obtain the depth self-attention feature maps, which can be formulated as in Equation (6). To restore the original feature space, $F_{sa}^{D}$ is performed by a linear operation.

(6) $$F_{sa}^{D}=\overset{N}{\underset{i=1}{\sum }}v\left(f_{i}\right)\times r_{j,i}$$

Similarity, the height self-attention is obtained by a height self-attention module and is present as $F_{sa}^{DH}$, while the width self-attention is present as $F_{sa}^{DHW}$. The attention features output by the axis attention fusion module are denoted as Equation (7) and the output of the axial attention mechanism is represented in Equation (8):

(7) $$F_{att}=F_{sa}^{DHW}+F$$

(8) $$F_{out}=F_{att}+F_{sc}$$

Since the method cannot be applied at the highest resolution feature representation (128 × 128 × 128), the AAM is employed at four lower resolutions. The number of attention heads and the dimension of each head, initially set to 4 and 16, were doubled as the resolution decreased.

### 3.3. The Deep Supervision (DS) Mechanism

A deep supervised learning scheme [27,28,29,30] is beneficial when training a CNN, enabling the network to collect gradients from the last and intermediate layers and propagate the error information layer by layer during the training process. DS, including three auxiliary output branches, is employed in the AABTS-Net, addressing the gradient’s vanishing and guiding the generation of better feature representations at each layer of the network. In detail, the auxiliary branch, achieved by three sigmoid outputs, is added in the middle three resolution levels (Figure 2). Notably, AABTS-Net contains four sigmoid outputs. Only the last output is the predicted segmentation image. The intermediate output, after being restored to the highest resolution level by up-sampling, is calculated in the auxiliary loss branch. The training loss of the network is the weighted sum of the loss of the intermediate and last outputs, giving each output a weight that decreases exponentially (divided by 2) with lower resolution, which makes higher-resolution outputs more weighted in the loss. The proposed deep supervision mechanism enables the network to generate better feature representation by collecting error information from more layers and by propagating misinformation to earlier layers, improving the expressiveness of the network and its segmentation performance.

### 3.4. The Hybrid Loss Function

The ratio between tumor area and background varies widely in the BraTS2021 training dataset, representing the imbalance of samples, making it difficult to precisely segment the lesion area [20,31]. The objective loss function, which also affects the segmentation performance of the network, is widely employed for handling the severe class imbalance of the datasets. A reasonable loss function should be designed for network optimization while retaining the complete information of the image, achieving the purpose of accurately segmenting small tumors. Many loss functions, such as Dice loss, cross-entropy loss, and focal loss, have been proposed for employment in segmentation, handling the class imbalance of samples. In this paper, a suitable hybrid loss function, combining the Dice and binary cross-entropy losses, is employed for solving class imbalance during the training process; it is computed at the lower-resolution auxiliary branch output and the final output (Equation (11)). Notably, the Dice loss (Equation (9)), referring to the batch-based Dice used here, is calculated by treating all samples in a batch as one larger sample. The common Dice loss, exhibiting gradient instability on highly imbalanced samples, is modified to the batch-based Dice to improve training stability. The cross-entropy (CE) loss function, aiming to handle issues arising from varying tumor sizes, is widely applied for increasing the sensitivity of segmenting tumor images. Since the activation function of the last layer is sigmoid, the corresponding output is the binary classification label. Then, a binary cross-entropy (BCE) loss function is employed (Equation (10)). Overall, the proposed hybrid loss function can guide our model to pay more attention to the tumor region to achieve ideal segmentation results, overcoming the problem of mapping tumors of different sizes and even small target tumors. These metrics are defined as follows:

(9) $${ℒ}_{Dice}=1-2\frac{\sum y_{p}y_{t}}{\sum y_{p}+y_{t}}$$

(10) $${ℒ}_{BCE}=-\lambda y_{t}\log\left(y_{p}\right)-\left(1-y_{t}\right)\log\left(1-y_{p}\right)$$

(11) $${ℒ}_{Total}={ℒ}_{H_{1}}+\alpha {ℒ}_{H_{2}}+\beta {ℒ}_{H_{3}}+\gamma {ℒ}_{H_{4}}$$

where $y_{t}$ represents the ground truth and $y_{p}$ represents the prediction result. α, β, and γ are the weight coefficient of the three auxiliary branches, and they are set to 0.5, 0.25, and 0.125, respectively (the higher the resolution, the greater the weight coefficient).

### 3.5. Experiments

#### 3.5.1. Datasets and Pre-Processing

In this work, the BraTS benchmark dataset [32,33,34] is applied to evaluate the proposed AABTS-Net. The BraTS dataset (Table 1) includes two sub-datasets, which comprise the training dataset and the validation dataset. In detail, the BraTS 2019 training dataset includes 335 cases, while the validation dataset includes 125 cases. The BraTS 2021 training dataset includes 1251 cases, and the validation dataset includes 219 cases. Each case of the dataset includes four MRI modalities, while a ground truth model included three different tumor classes (non-enhanced and necrotic tumor cores—label 1, peritumoral edema—label 2, and GD-enhanced tumors—label 4). The ground truth annotations of the training dataset are publicly available and are created by expert neuroradiologists. Since the annotations of the validation dataset are unavailable, we tested the performance by uploading the predicted segmentation results to the official platform provided by the organizer.

All cases in the datasets, having undergone basic preprocessing by the organizers, were interpolated to $1\times 1\times 1\;{\mathrm{mm}}^{3}$, co-registered, and skull-stripped. On this basis, we only performed relatively simple data preprocessing. First, the 3D raw data were cropped to a nonzero region. One case in the BraTS dataset showed relatively more black background (gray value 0). Since this area has no information, cropping these areas did not affect the final segmentation result. However, the process can significantly reduce the image size, avoid useless calculations, and improve computational efficiency. Specifically, it is necessary to find a minimum three-dimensional bounding box in the image, assign the value outside the bounding box area as 0, and use this bounding box to crop the raw data. Moreover, we used normalization in a standard space for each case in the dataset. In detail, employing a z-score ($X_{z-score}=\frac{X-\mu }{\sigma }$, where μ and σ are mean and standard deviation) for the normalization operation accelerates the training. Notably, under the conditions that the image size is reduced by more than 1/4, the normalization operation is only performed on the non-zero regions. Dynamic data augmentation, a technique intended to prevent overfitting while training a CNN, is also employed, improving the robustness and the generalization ability of the AABTS-Net. Specifically, five types of data augmentation, comprising random rotation, random scaling, elastic deformation, gamma augmentation, and additional brightness enhancement, with a probability of 0.3, were applied during the training. The random scaling factor is (0.65, 1.6), using a single scaling factor for each axis.

#### 3.5.2. Evaluation Metrics

Three evaluation metrics, including Dice similarity coefficients (DSC), the Hausdorff distance (HD), and Sensitivity, were employed to evaluate the performance of the models. The DSC is a widely considered criterion, quantitatively assessing the 3D predicted segmentation of the model. It provides the similarity or overlap of two samples, with a value in the range [0–1], and is defined as in Equation (12). The higher the value, the more similar the two samples are, indicating more accurate performance between the predicted segmentation and manual segmentation. Additionally, when affected by uncertain factors, such as shape, location, and size, the model performance also depends on the accuracy of segmenting tumor boundaries. Considering the fact that the DSC is sensitive to the inner filling of the mask, the HD, computing the distance between the surface of the prediction regions and the ground truth, is employed for assessing the performance of the boundary segmentation. The HD is defined as follows (Equation (13)). Sensitivity measures the ability of the model to segment the region of interest by calculating the proportions of true positives in the predicted segmentation results and ground truth and is given by Equation (14).

(12) $$SC=\frac{2TP}{2TP+FP+FN}$$

(13) $$HD\left(G,P\right)=max\;\left\{sup_{s\epsilon S}inf_{r\epsilon R}d\left(s,r\right),\;sup_{r\epsilon R}inf_{s\epsilon S}d\left(s,r\right)\right\}\;$$

(14) $$Sensitivity=\frac{TP}{TP+FN}$$

where FN, FP, and TP are the number of false negative voxels, false positive voxels and true positive voxels. Here, d(·,·) is the function that computes the distance between points s and r, while sup represents the supremum and inf denotes the infimum; s and r denote the points on surface S of the ground-truth regions and surface R of the predicted regions, respectively.

#### 3.5.3. The Training Details

The model, established on the Pytorch framework, was implemented using a single NVIDIA Tesla P100 GPU, with 16 GB of memory. The Adam optimizer [35], an essential optimization algorithm, was employed for gradient descent. The initial learning rate of 10^−4^ is progressively multiplied by ${\left(\frac{1-epoch}{\max epoch\;}\right)}^{0.9}$ during the training. The k-fold cross-validation procedure was used to estimate the performance of the model on new data. In our experiments, five-fold cross-validation is set up during the training of the model. Specifically, the training dataset was divided (randomly) into five parts, training for four parts and testing for one part. Each epoch consisted of 250 mini-batches, and training did not end until the maximum epoch (300) was achieved (Table 2).

## 4. Results

### 4.1. Comparison with State-of-the-Art Methods

#### 4.1.1. The Experiment Results on the BraTS 2019 Dataset

**Figure 4:**
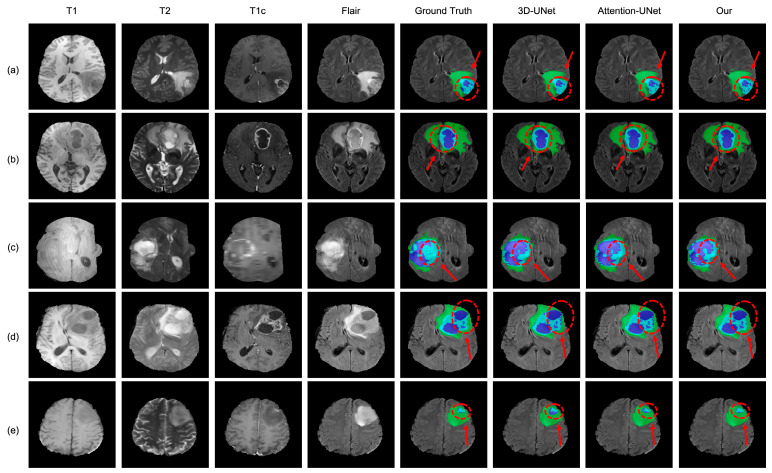
The visualized segmentation results of the proposed AABTS-Net and other methods. (**a**–**e**) represents different visualized cases. From left to right: T1, T2, T1c, Flair, Ground truth, the result segmented by 3D-UNet, the result segmented by Attention-UNet, and the result segmented by our model.

The BraTS 2019 dataset was applied to evaluate the effectiveness and robustness of the proposed AABTS-Net. The qualitative comparison is shown in Figure 4, from left to right: four MRI modalities, corresponding ground truth, 3D-UNet, Attention-UNet, and our method. From Figure 4, it can be seen that these methods can roughly segment the tumor region. However, detailed information on the tumor boundary is missing. In detail, the segmentation results generated by 3D-UNet have an obviously inaccurate tumor boundary. The segmentation results generated by Attention-UNet improved the segmentation, but the tumor boundary still demonstrates the problem of missing information. The segmentation results produced by our proposed AABTS-Net are more accurate and are similar to the ground truth results, effectively refining the boundaries of brain tumors and enhancing the detailed features.

In addition, we evaluated the model on the BraTS 2019 validation dataset. The BraTS 2019 validation dataset contains 125 cases. It is worth noting that the BraTS 2019 training dataset and the BraTS 2019 validation dataset are two independent datasets provided by the organizer. Since the ground truth of the BraTS 2019 validation dataset is unavailable, we uploaded the segmentation results to the official website and obtained the quantitative results from the platform. We also performed comparative experiments (Table 3) between our method and other state-of-the-art methods. From Table 3, it is clear that our method achieves DSC values of 0.777, 0.838, and 0.911, at ET, TC, and WT, respectively, which values are higher than those of other methods. The DSC increased by 3.7% to 24.7% in ET, and the DSC increased by 0.8% to 15.6% and 1% to 6.9% in TC and WT, respectively. Compared with the other methods, our method shows great superiority in both metrics, with significant improvements. The comparison results show that our method achieves superior performance.

#### 4.1.2. The Experiment Results on the Brats 2021 Dataset

Comparison of the segmentation performance between our method and other state-of-the-art methods is conducive to analyzing the advantages and disadvantages of the model and achieving improvements in segmentation performance. The predicted results (see Table 4) on the BraTS 2021 validation dataset, showing the three tumor subregions, were analyzed by employing two evaluation metrics for assessing the performance of the model. As shown in Table 4, the DSC at three tumor sub-regions was significantly distinct for the DSC 0.830, 0.861, and 0.922 at ET, TC, and WT, showing a clear upward trend that represents an accurate segmentation: the larger the value, the more precise the segmentation. The HD, being different from the DSC, posed a downward trend at three tumor sub-regions, with the maximum value of 17.728 observed at ET and the minimum value of 3.996 observed at WT, indicating that the AABTS-Net method can precisely segment the boundary of the tumor. Compared with other methods [20,42,43,44,45,46], the DSC increased by 0.4% to 5.9% in WT. The DSC increased by 1.3% to 13.7% and 1% to 14.6% in ET and TC, respectively. This demonstrates that our method achieved a promotive performance in terms of tumor sub-region segmentation. In addition, compared with other state-of-the-art UNet-based models [24,47,48], our model still achieved promising segmentation performance by analyzing the different metrics. Our model has fewer parameters and refines the boundaries of the tumor subregions by enhancing the local and global feature information.

**Figure 5:**
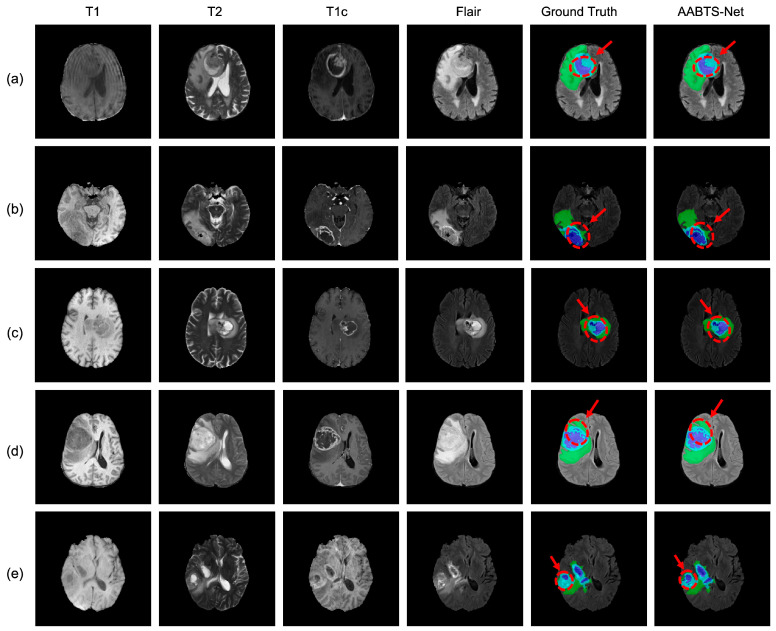
The visualized segmentation results of the proposed AABTS-Net. (**a**–**e**) represents different visualized cases. From left to right: T1, T2, T1c, Flair, Ground truth and the result segmented by AABTS-Net.

Furthermore, we segmented the cases (shown in Figure 5) selected from the BraTS 2021 dataset, visualizing the predicted segmentation results. Specifically, the first case segmented by our model retained detailed information and showed many similarities with the GT. The predicted segmentation results of AABTS-Net were not significantly affected, as the input MRI modality is blurred, as shown in Figure 5 (the fourth row). In conclusion, the results predicted by AABTS-Net, effectively refining the boundaries of the tumor subregions and enhancing the local and global feature information, are not significantly different from those of the GT. This phenomenon suggests that the AABTS-Net model can learn better feature representations to localize tumors and guide the model to produce accurate segmentation results. In general, the AABTS-Net model achieved accurate brain tumor segmentation and can be employed to assist doctors in diagnosis.

### 4.2. The Analysis of Key Components

#### 4.2.1. Analysis of the Axial Attention Mechanism

**Figure 6:**
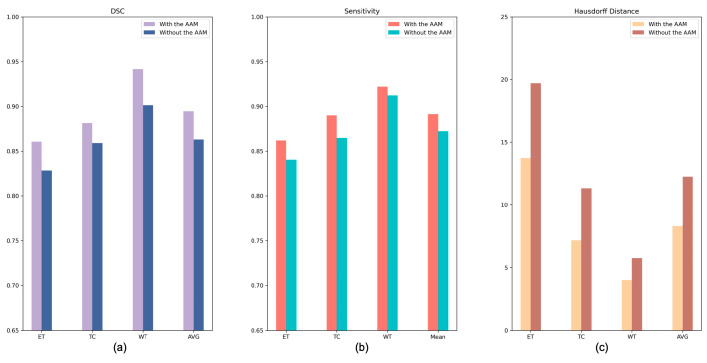
The performance indexes of the proposed AABTS-Net and the AABTS-Net without AAM. (**a**) represents the DSC value, (**b**) represents the Sensitivity value and (**c**) represents the HD value.

**Figure 7:**
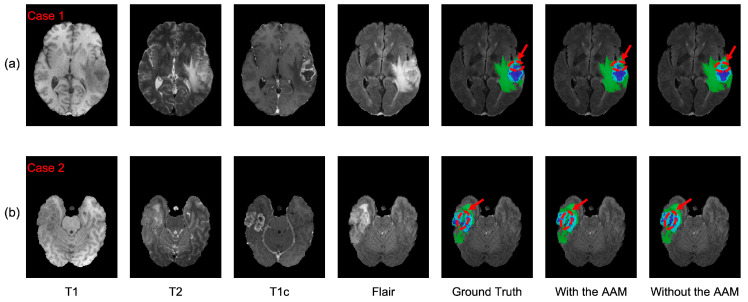
The visualized segmentation results of the proposed AABTS-Net and the AABTS-Net without AAM. (**a**,**b**) represent the different visualized cases. From left to right: T1, T2, T1c, Flair, Ground truth, the result segmented by AABTS-Net with the AAM and the result segmented by AABTS-Net without the AAM.

**Figure 8:**
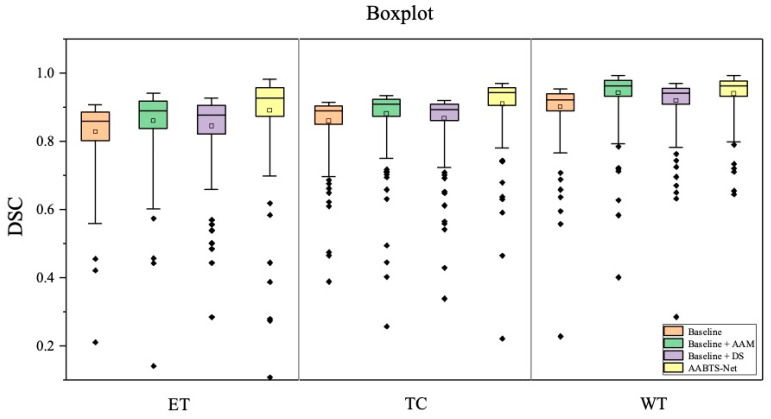
The DSC boxplot of the ablation study in three tumor regions.

To verify the effectiveness of the AAM, we conducted ablation experiments (see Table 5) to compare the proposed AABTS-Net with the same network without employing the AAM. The DSC of subregions ET, TC, and WT exhibited an upward trend. The reason for this may be that the structural distribution of the ET region is complex, and the model found it difficult to distinguish between the edema region and the tumor core and the surrounding area, enhancing the tumors. Segmentation of the different tumor subregions improved as the AAM was added to the network. The network with the AAM improved DSC by 4.6%, 2.6%, and 3.9% in the WT, TC, and ET regions, respectively. According to the results (Figure 6 of the two models on the three metrics, the corresponding DSC and Sensitivity values were increased significantly by applying the AAM to the model. Furthermore, we visualized the segmentation results generated by AABTS-Net and AABTS-Net, removing the AAM. From the predicted segmentation results (Figure 7) of the two cases, it can be seen that both models can segment the WT region. However, the AABTS-Net’s removal of the AAM, segmenting the ET and TC, obtained a rough edge (red arrows). By removing the AAM, the model’s ability to recognize fuzzy boundaries was weakened, thereby reducing the segmentation accuracy of ET and TC. The segmentation results predicted by AABTS-Net were similar to ground truth, which shows that the proposed AAM improved the segmentation performance. In addition, we have drawn a DSC boxplot to analyze the contribution of the AAM. From Figure 8, we observed that the AAM improved the result significantly in terms of the different tumor regions. In general, the values of the three metrics and the visualized result, showing the better values of the tumor subregions and the predicted result with more detailed information, indicated that the segmentation performance was improved by employing the AAM. This can be attributed to AAM’s ability to model local–global feature information and the resulting enhanced capability of boundary recognition.

#### 4.2.2. Analysis of the Deep Supervision Mechanism

**Figure 9:**
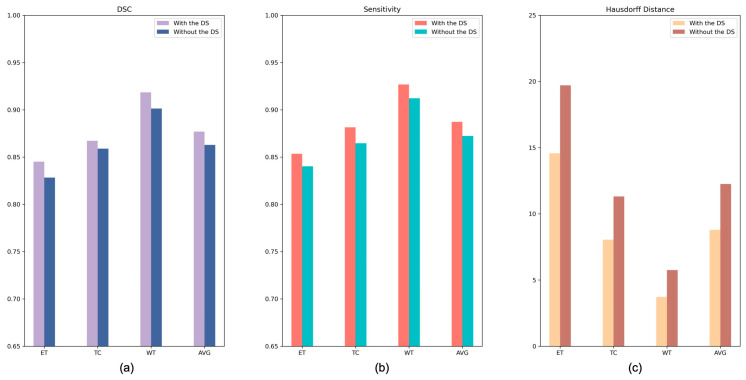
The performance indexes of the proposed AABTS-Net and the AABTS-Net without DS. (**a**) represents the DSC value, (**b**) represents the Sensitivity value and (**c**) represents the HD value.

**Figure 10:**
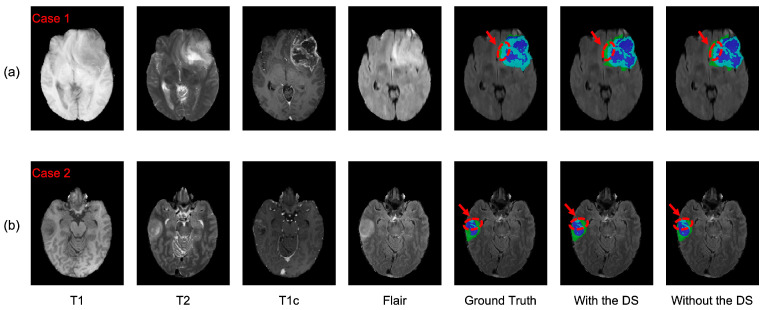
The visualized segmentation results of the proposed AABTS-Net and the AABTS-Net without DS. (**a**,**b**) represent the different visualized cases. From left to right: T1, T2, T1c, Flair, Ground truth, the result segmented by AABTS-Net with the DS and the result segmented by AABTS-Net without the DS.

To evaluate the contribution of the DS to the AABTS-Net, we conducted comparative experiments on the BraTS2021 dataset (Table 5). Specifically, Figure 9 shows the experimental results of AABTS-Net with and without the DS on three metrics (DSC, HD, and Sensitivity). It is clear that the DSC and Sensitivity are significantly increased in the scenario where DS is applied to AABTS-Net, which indicates that DS assists the network, to improve the segmentation performance. The DSC value increased by 2.0%, 0.9%, and 2.1% in the WT, TC, and ET regions, respectively. From the WT region, the DSC value (see Table 5) after adding DS was smaller than that after adding AAM. This was mainly due to the fact that the DS was employed for gradually refining the segmentation and retaining more detailed tumor information. However, this detailed information has less of a contribution than the tumor masses to the DSC value. The HD value (the smaller the HD value, the better the performance) decreased by 35.2%, 28.9%, and 26.0% in the WT, TC, and ET regions, respectively. This large reduction in the HD value also indicates that more accurate edge segmentation and more detailed information can be obtained when using DS. The Sensitivity value increased by 1.6%, 2.0%, and 1.7% in the WT, TC, and ET regions, respectively. From Figure 8, it can be seen that the model with the DS showed better segmentation results in the WT and ET regions. Figure 10 shows the segmentation results produced by the AABTS-Net and the AABTS-Net without the DS. From Figure 10, it can be seen that the tumor regions (red arrows) segmented by AABTS-Net have more similarities with the ground truth. This demonstrates that the DS not only guides the model to generate better feature representations from multi-modality but also makes full use of the different feature representations.

#### 4.2.3. Analysis of the Different Loss Functions

**Figure 11:**
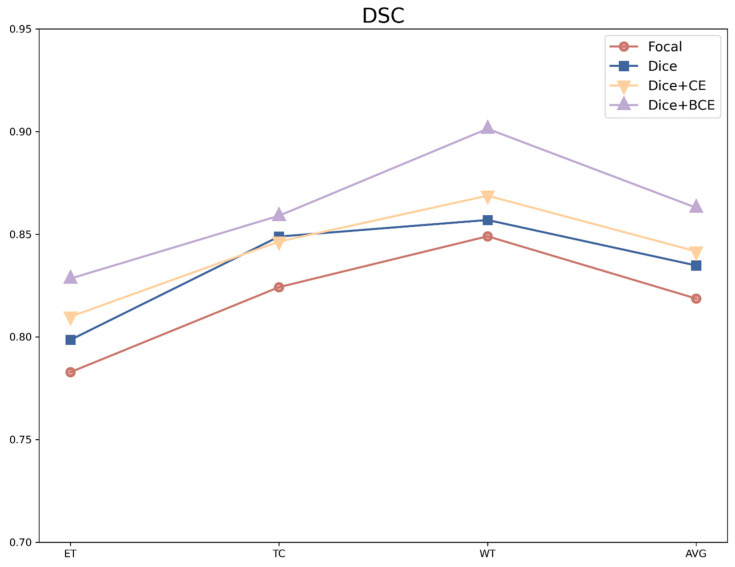
The DSC values for the different losses.

Additionally, for class-imbalanced data, choosing an appropriate loss function fulfills an important role in segmentation accuracy. The DSC variations of different loss functions were observed. However, using Dice alone is not conducive to backpropagation, and it is easy to cause parameter oscillation during training, which is not conducive to convergence. Therefore, we considered applying a combined loss function for training. As shown in Figure 6, the segmentation performance metrics at four loss functions (i.e., Focal, Dice, Dice + CE, and Dice + BCE) fluctuated significantly, and three notable changes occurred when segmenting the different tumor subregions. The DSC of the ET sub-region in four loss functions was relatively low. However, the DSC values increased at the Focal, Dice, Dice + CE, and Dice + BCE loss functions. In detail, the average DSC values increased from 0.819 to 0.863 at the Focal and the Dice + BCE loss functions, respectively. In addition, the maximum DSC values of all loss functions are shown, with the DSC values of 0.828, 0.859, and 0.901 in the ET, TC, and WT subregions, respectively. From the Dice + BCE loss function, the DSC values at ET, TC, and WT sub-region also increased significantly. According to the average DSC values (see Figure 11), the DSC values of all loss functions (Focal, Dice, Dice + CE, and Dice + BCE) exhibited an upward trend, while the DSC value of the Dice + BCE loss function was significantly higher than the other loss functions. Overall, the combination of Dice and BCE, which achieved the best segmentation results, was employed for optimizing the network during the training process. The segmentation accuracy of improvement can benefit from the BCE loss function and the modified batch-based Dice loss function, as when handling the class imbalance from the BCE loss function and improving the backpropagation from the combination loss function. Moreover, the hybrid loss function, which played a vital role in segmentation performance, was an innovative and effective strategy for solving the segmenting of tumors of different sizes.

### 4.3. Analysis of the Bad Tumor Segmentation Results

**Figure 12:**
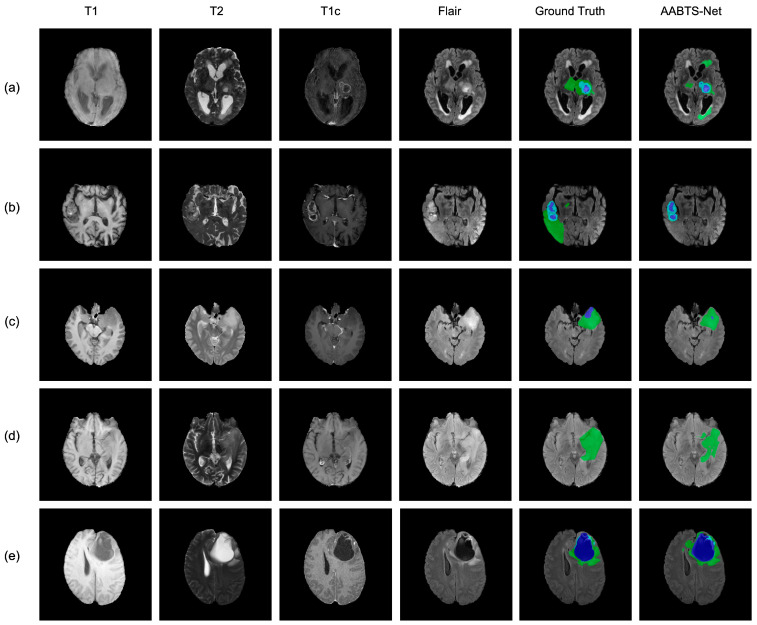
The visualized bad tumor segmentation cases of the proposed AABTS-Net. (**a**–**e**) represents different visualized cases. From left to right: T1, T2, T1c, Flair, Ground truth, the result segmented by AABTS-Net.

To further analyze the segmentation performance, we selected five bad segmentation cases and visualized the segmentation results of the proposed AABTS-Net. As shown in Figure 12, each row contained four different modalities for a case, the ground truth, and the segmentation result generated by the AABTS-Net. From the first two cases, we observed that the ET and TC regions segmented by the AABTS-Net are similar to the ground truth. However, the similar characteristics of normal and tumor regions led to the WT region exhibiting over-segmentation. From the third and fourth cases, we observed that the tumor regions generated by the AABTS-Net were significantly different from the ground truth. This may be due to the ambiguity of the MRI modality, making it difficult for the network to identify tumor regions from the MRI modality. In addition, we quantified the cases of bad tumor segmentation in terms of the DSC (Table 6). By analyzing the bad segmentation cases, we realized that the quality of the MRI modality can affect the segmentation performance. Therefore, we considered the impact of data quality on network performance when designing the model and must collect more clear data in a different way to further improve the accuracy by optimizing the tumor margins.

## 5. Discussion

In this paper, AABTS-Net, an axial attention convolutional neural network, was established for multi-modality MRI brain tumor segmentation. The axial attention mechanism, employed to decrease the high computational complexity of applying the attention mechanism to three-dimensional medical images, capturing plenty of semantic information, achieved accurate segmentation and improved segmentation performance. The deep supervision mechanism guided the AABTS-Net to generate better feature representations, handling the problem of the vanishing gradient easily and improving the stability of the network. The hybrid loss model was employed for dealing with the class imbalance of the data. The AABTS-Net exhibited superior performance on the BraTS 2021 validation dataset, with DSC values of 0.830, 0.861, and 0.922, and HD values of 17.728, 11.178, and 3.996, respectively, at the three tumor subregions (ET, TC, and WT). Furthermore, the quantitative and qualitative experiments demonstrated the accuracy of the AABTS-Net. Experiments using two datasets demonstrated the effectiveness of our model compared to previous work. Our model achieved better robustness and accuracy, which indicated that the AABTS-Net can be employed to assist doctors in diagnosis. However, we observed that our model has some disadvantages in terms of TC region segmentation when we compared our method with the Extending nn-UNet in the context of the HD metric. To resolve this issue, we applied a simple feature extraction method that ignored the shallow feature fusion. In the future, we will consider employing a deeper CNN to extract the features and applying a more flexible feature fusion strategy to improve the accuracy by fusing multi-modality feature representations.

## Figures and Tables

**Table 1:** A description of the datasets in this work.

Dataset	Training Dataset	Validation Dataset *
BraTS 2019	335 cases	125 cases
BraTS 2021	1251 cases	219 cases

* Without ground truth.

**Table 2:** The training parameter of the proposed AABTS-Net.

Name	Parameter
Batch_size	2
Epoch	300
Learning rate	0.0001
GPU memory	16 GB
Patch size	128 × 128 × 128
Optimizer	Adam
Framework	Pytorch

**Table 3:** Objective performance comparison with the state-of-the-art methods on the BraTS2019 validation dataset, in terms of their DSC and HD values. (Bold font indicates best results.)

Methods	DSC				HD			
	ET	TC	WT	AVG	ET	TC	WT	AVG
3D UNet [36]	0.709	0.725	0.874	0.769	5.062	8.719	9.432	7.738
Attention UNet [37]	0.760	0.772	0.888	0.807	5.202	7.756	8.258	7.072
Shi et al. [38]	0.691	0.770	0.887	0.783	5.888	12.192	21.190	13.090
Zhao et al. [39]	0.702	0.800	0.893	0.798	4.766	6.472	5.078	5.439
Lorenzo et al. [40]	0.663	0.751	0.890	0.768	/	/	/	/
Ahmad et al. [41]	0.623	0.758	0.852	0.744	8.468	10.674	9.008	9.383
Our Method	**0.777**	**0.838**	**0.911**	**0.842**	**3.246**	**6.028**	**3.988**	**4.421**

**Table 4:** Objective performance comparison with the state-of-the-art methods on the BraTS2021 validation dataset, in terms of their DSC and HD values. (Bold font indicates best results.)

Methods	DSC				HD			
	ET	TC	WT	AVG	ET	TC	WT	AVG
ESA-Net [20]	0.812	0.852	0.907	0.857	26.470	13.830	5.940	15.413
3D CMM-Net [42]	0.732	0.751	0.874	0.786	35.001	24.638	10.161	23.267
SwinBTS [43]	0.832	0.848	0.918	0.866	**16.030**	14.510	3.650	11.397
Singh et al. [44]	0.730	0.760	0.870	0.787	30.500	14.700	6.290	17.163
Akbar et al. [45]	0.780	0.807	0.891	0.826	25.820	21.170	11.780	19.590
Singh et al. [46]	0.753	0.808	0.899	0.820	21.800	12.500	6.450	13.583
Extending nn-UNet [47]	**0.845**	**0.878**	**0.928**	**0.884**	20.730	**7.623**	**3.470**	**10.608**
E1D3-UNet [48]	0.818	0.863	0.923	0.868	18.240	9.620	4.340	10.733
Bitr-UNet [24]	0.819	0.843	0.910	0.857	17.847	16.689	4.508	13.015
Our Method	0.830	0.861	0.922	0.871	17.728	11.178	3.996	10.967

**Table 5:** The ablation studies of the different methods on the BraTS 2021 training dataset (1251 cases). All experiments were performed via 5-fold cross-validation on the training cases (no external data were used). (Bold font indicates best results.)

Methods	DSC				HD				Sensitivity			
	ET	TC	WT	AVG	ET	TC	WT	AVG	ET	TC	WT	AVG
Baseline	0.828	0.859	0.901	0.863	19.703	11.310	5.743	12.252	0.840	0.865	0.912	0.872
Baseline + AAM	0.860	0.881	**0.942**	0.894	13.728	7.178	3.996	8.301	0.862	0.890	0.922	0.891
Baseline + DS	0.845	0.867	0.919	0.877	14.583	8.042	3.723	8.783	0.854	0.882	0.927	0.887
AABTS-Net	**0.890**	**0.909**	0.940	**0.913**	**10.132**	**7.132**	**3.343**	**6.869**	**0.905**	**0.915**	**0.952**	**0.924**

**Table 6:** The DSCs of the bad segmentation cases.

Cases	DSC			
	ET	TC	WT	AVG
(a) BraTS2021_00493	0.885	0.924	0.270	0.693
(b) BraTS2021_00494	0.964	0.990	0.730	0.895
(c) BraTS2021_01666	0	0.738	0.916	0.551
(d) BraTS2021_01179	1.000	0	0.769	0.590
(e) BraTS2021_01293	0.541	0.929	0.874	0.781

## Data Availability

The datasets are provided by BraTS Challenges (2019, 2020, and 2021) and are allowed for personal academic research. The specific link to the dataset is https://ipp.cbica.upenn.edu/ (accessed on 3 November 2021).
